# Effects of pre-sleep protein supplementation on plasma markers of muscle damage and inflammatory cytokines resulting from sprint interval training in trained swimmers

**DOI:** 10.1080/15502783.2023.2244478

**Published:** 2023-08-06

**Authors:** Cairong Wu, Jie Deng, Chengli Gao

**Affiliations:** a Zhengde Polytechnic College, Department of Public Education, Nanjing, Jiangsu, China; b Adamson University, Graduate School, Metro Manila, Philippines; c Nanjing University of Aeronautics and Astronautics, Department of Physical Education, Nanjing, Jiangsu, China; d Sanjiang University, Department of Physical Education, Nanjing, Jiangsu, China

**Keywords:** Intermittent exercise, muscle damage, dietary protein, supplement, cytokine, conditioning

## Abstract

**Background:**

Pre-sleep protein has been shown to improve muscle recovery overnight following exercise-induced muscle damage. Whether such an approach affects recovery from sprint interval training (SIT) has yet to be elucidated. This study examined the effects of protein supplementation every night before sleep on early (45 min post-SIT) and late (24 and 48 h after SIT) responses of creatine kinase (CK) and inflammatory cytokines, including interleukin-6 and 10 (IL-6 and IL-10) and tumor necrosis factor-alpha (TNFα).

**Methods:**

Twenty trained swimmers underwent a 2-week in-water swimming SIT (two sets of 12 × 50-m all-out swims, interspersed by 1:1 recovery between each sprint and 3 min of rest between sets) and were randomized to two intervention groups receiving either 0.5 g kg^−1^ day^−1^ protein beverage (PRO) or the same amount of carbohydrate (CHO) preceding going to bed every night. For initial and final training sessions, CK and cytokine responses were analyzed at different time points, including resting, immediately after completion, 45 min post-SIT, and 24 and 48 h after SIT.

**Results:**

CK concentrations elevated from resting point to 24 and 48 h post-SIT for both PRO and CHO groups (*p* < 0.05). In both training groups, the peak levels of IL-6 and 10 were observed 45 min post-SIT on both occasions. TNFα levels significantly elevated from rest to immediately after SIT (*p* < 0.001) and returned to values equivalent to the baseline afterward in both groups and on both occasions. In both groups, swimming SIT also switched the cytokine response 48 hours after exercise to an anti-inflammatory status by decreasing the ratio of IL-6 to IL-10 (*p* < 0.04) in the last training session.

**Conclusions:**

Pre-sleep protein ingestion failed to ameliorate blood markers of muscle damage. The late anti-inflammatory profile of cytokines and exercise-induced muscle damage improved after two weeks of swimming SIT with either protein or carbohydrate ingestion before sleep.

## 1. Introduction

During annual training cycles, swimmers periodically undertake high-intensity training interventions. Sprint interval training (SIT) is one of the frequently used intensive interventions by swimmers [1–3] which can promote power output as well as physiological adaptations associated with the central and peripheral components of aerobic fitness [4]. Typically SIT sessions involve interval periods of effort completed at the extreme exercise domain, usually consisting of 4 to 12 bouts of ~ 10 to 30 s self-paced, all-out efforts with varying recovery intervals [3].

The supramaximal intensity of SIT undertaking the musculoskeletal system to great strain can elicit muscle damage [5], resulting in muscle soreness, inflammatory response, and loss of muscle force. The symptoms mentioned may persist following such intensive exercise bouts for hours and days and compromise subsequent physical activity at the next exercise sessions [6]. It is crucial to ensure efficient muscle recovery during such periods to sustain training intensity and prevent the risk of overuse injuries. Thus, elucidating nutritional interventions to improve muscle recovery after SIT-induced strain would be particularly important to maintain training intensity and enhance the conditioning quality.

Studies have revealed that protein ingestion before, during, or following exercise can improve recovery and training adaptations while minimizing exercise-induced catabolism [7–9]. Beneficial effects of protein feeding methods to optimize recovery through attenuation of circulatory markers indicating disruption of the sarcolemma [10,11] and improvements in markers of immune function [6,12] as well as protein balance [13,14] have been unveiled. Traditional protein feeding strategies involve its consuming during and immediately around training sessions [15].

Emerging evidence suggests that there may be potential advantages to consuming protein just before sleeping overnight [13,16–19]. While the majority of studies supporting the positive effects of pre-sleep protein supplementation have primarily used casein as the protein source, recent findings by Trommelen and colleagues [20] suggest that there is no significant difference in the muscle protein synthesis in response to whey and casein protein when consumed before sleep. Studies have shown that providing the body with protein before sleep increases the availability of plasma amino acids overnight, leading to enhanced muscle protein synthesis while sleeping [13,14]. However, it is unclear if pre-sleep protein supplementation may affect SIT-induced increases in markers of sarcolemma disruption and inflammatory cytokines. There is also a lack of information on how SIT affects the body’s anti-inflammatory status over an extended period. Therefore, we undertook this study to investigate a) how one session and a 2-week swimming SIT affect markers of inflammation and muscle damage and if increasing protein intake through protein supplementation before sleep may affect these responses. We hypothesized that providing the body with a whey protein supplement every night before bedtime would diminish post-exercise muscle damage and improve the cytokine response by shifting its status to an anti-inflammatory profile.

## 2. Materials and methods

### 2.1. Participants

Table 2 presents the characteristics of the participants. Twenty male trained swimmers (mean ± SD; age = 23.1 ± 2.7 years; height = 180.7 ± 6.3 cm; body mass = 81.6 ± 3.1; body fat = 11.2 ± 1.5%; years of experience = 9 ± 5) sign their informed consent and volunteered to participate. Participants’ training habits were documented using a questionnaire, and based on the framework suggested by McKay and colleagues [21], they were classified as trained athletes. The study received ethical approval from the research ethical board of the Adamson University and all procedures were completed per the ethical principles of the World Medical Association (WMA).

**Table 1.** Training history two months prior to participation in this study.

	Swimming				Endurance					Other		
	PRO		CHO	*p*	PRO		CHO			PRO		CHO
	Training history											
Duration (h wk^−1^)	8.3 ± 4.3		7.8 ± 3.2	0.71	3.9 ± 2.8		2.4 ± 2.3			2.1 ± 1.9		3.4 ± 3.1
Distance (km wk^−1^)	26 ± 11		23 ± 9	0.64								

Data were based on questionnaires and logs. Statistical analysis for training history was based on actual swimming sessions only. Endurance training covers a range of disciplines (i.e. running and cycling).

**Table 2.** Characteristics of the participants.

		Characteristics	
	PRO	CHO	ALL
Age (years)	23.7 ± 2.5	22.6 ± 2.9	23.1 ± 2.7
Body mass (kg)	81.4 ± 3.3	81.9 ± 2.9	81.6 ± 3.1
Body fat (%)	10.9 ± 1.5	11.6 ± 1.6	11.2 ± 1.5
Height (cm)	180.7 ± 5.9	180.8 ± 6.7	180.7 ± 6.3
Energy (Mj day^−1^)	12.6 ± 0.3	12.8 ± 0.4	12.8 ± 0.4
Protein (%energy)	13.3 ± 1.1	13.4 ± 0.8	13.4 ± 0.8
Protein (g kg^−1^ day^−1^)	1.26 ± 0.05	1.36 ± 0.07	1.36 ± 0.07
Carbohydrate (%energy)	51.3 ± 4.6	52.6 ± 2.5	52.6 ± 2.5
Fat (%energy)	35.3 ± 1.0	33.8 ± 1.4	33.8 ± 1.4

Values are means ± SD.

### 2.2. Experimental design

**Figure 1.**
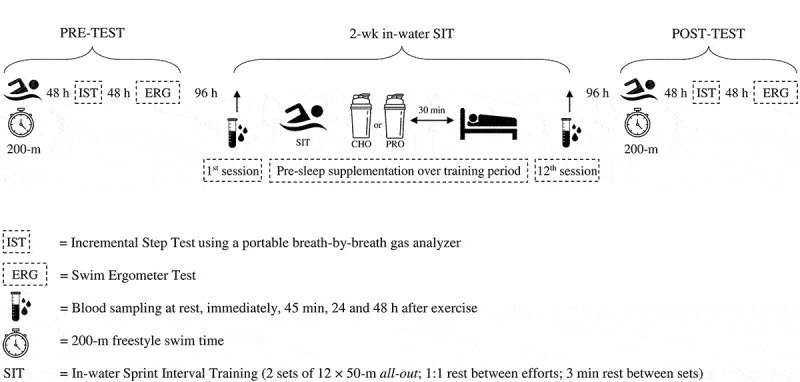
Overview of experimental protocol.

This double-blind, randomized-controlled intervention study consisted of a pretest, training intervention, and posttest (Figure 1). Following screening for unknown diseases, participants were paired based on their physiological and physical characteristics and training history (Table 1). Then, participants of a determined pair were randomized to either the protein (PRO) or carbohydrate (CHO) group. The experiment was initiated two weeks after the participant recruitment to ensure the washing out of previously consumed supplements.

Before and after the training, participants performed a discontinuous incremental step test to evaluate aerobic power and related physiological parameters. Participants’ peak and average power output (PPO & APO) were assessed by completing an *all-out* 6 s and 30 s sprint test using a swim ergometer (VASA, Essex Junction, VT, USA). Also, 200-m freestyle swimming performances were determined on a particular day. Body composition was analyzed using a bioelectric impedance analyzer (Inbody 570, Biospace Co, Ltd., Korea). Participants were advised to abstain from alcohol and take supplements during the study and avoid strenuous exercise for at least 24 h period preceding testing sessions. A gap of 48 h separated tests to ensure adequate recovery time. 96 h after finishing the baseline measurements, the first training session of the 2-week SIT program was initiated. During the first and last training sessions, blood samples were collected at different time points, including resting, immediately after completion, 45 min post-SIT (early response), and 24 and 48 h after SIT (late response). 96 h after finishing the last session of the SIT, all participants underwent the same testing battery as the pre-training assessment in the same sequence and under comparable conditions.

#### 2.2.1. Measurements

##### 2.2.1.1. Discontinuous incremental step test

Each participant completed an individualized intermittent incremental protocol with speed incrementing by 0.05 m · s ^−1^ during seven 200-m steps, including 30 s breaks between each stage until reaching exhaustion. The predefined speed of the initial step was determined by subtracting seven increments of pace from the athlete’s best time in the 400-m front crawl. A flashing light visual pacer (TAR.1.1, GBK-electronics, Aveiro, Portugal) placed in the pool base was used to ensure a controlled swimming speed during the test. A portable gas collection system (K4b^2^, Cosmed, Rome, Italy) connected the athlete using a respiratory snorkel with low hydrodynamic resistance and a valve system (AquaTrainer®, Rome, Italy) continuously analyzed pulmonary gas exchange and measured physiological parameters. VO_2_peak was confirmed based on the following criteria: 1) a level off in VO_2_, despite increasing the intensity of swimming, and 2) RER ≥ 1.1, peak HR > 90% of maximum HR predicted for the age, blood lactate acid concentrations equal to or exceeding 8 mmol·l^−1^, and visible signs of exhaustion [22,23]. All tests were completed in a swimming pool (25-m length,1.9-m depth, and 28°C water temperature).

### 2.3. Determination of PPO and MPO using swim ergometer

To evaluate peak and average power output, swimmers completed two maximal tests of 6 s and 30 s on the swim ergometer. Following a 5 min warm-up consisting of ten incremental pulls from low to submaximal intensity, participants were instructed to perform maximal sprint at zero resistance with one h passive recovery between trials. A zero resistance was set for the test to ensure pulling is implemented with maximal velocity entire the test [2].

### 2.4. 200-m swimming performance

After a standardized warm-up provided by CuencaFernández and colleagues [24], participants performed a short-course 200-m freestyle swimming performance in a testing environment under swimming competition conditions. Using a stopwatch (3X-100 M, Tracy, California, USA), two experienced timekeepers measured the time of the trial.

### 2.5. Blood sampling

10 ml of venous blood was collected using venipuncture at different time points, including resting, immediately after completion, 45 min post-SIT (early response), and 24 and 48 h after SIT (late response). The blood sample was spun at 4ºC for 15 min at 1000 × g within 30 min of collection, and the separated plasma was stored at −80ºC and was analyzed subsequently. CK concentrations (MyBioSource, MBS269244; San Diego, California, USA), Interleukin 6 and 10 (IL-6 and IL-10), and tumor necrosis factor-alpha (TNFα) (Elabscience, E-EL-H6156, 6154, and 0109, respectively; Houston, Texas, USA) were analyzed using ELISA assays per manufacturer instructions. The coefficients for variation were <10%, and the sensitivities were 0.06 ng/mL, 0.93 pg/mL, 0.94 pg/mL, and 4.69 pg/mL, respectively.

### 2.6. Training protocol

The training period was two weeks, and participants performed six sessions/week of SIT consisting of two sets of 12 × 50-m all-out swims from a wall push with 3 min of rest between sets. To prevent a decline in the volume of O_2_ consumption during recovery, a short work-to-recovery ratio of 1:1 was used between each sprint, allowing swimmers to swim close to VO_2_peak during sets [5,25]. The recovery period involved passive rest (i.e. swimmers were asked to avoid swimming). Each in-water SIT session began with a 10 min warm-up, followed by two series of 12 × 50-m *all-out* swimming with 3 min relief between series, and was completed with 10 min cool-down. No additional training was allowed.

### 2.7. Dietary control

Resting metabolic rate (RMR) was measured using an indirect calorimeter (Fitmate Pro, Cosmed, Rome, Italy) and according to the recent guidelines [26]. Total daily energy expenditure was determined from RMR × daily physical activity level beside training + estimated energy expenditure during exercise. The estimated individual’s energy expenditure was used to determine individual diet’s caloric value. During the training period, all participants ingested 1.3 g kg^−1^ day^−1^ protein via standardized diet. Participants receiving the whey protein supplement ingested an additional 0.5 g kg^−1^ day^−1^ of protein before sleep. Research conducted by Mamerow and colleagues [27] revealed that evenly distributing daily protein intake across three main meals leads to increased muscle protein synthesis rates over 24 h, in contrast to an imbalanced distribution where most protein is consumed during dinner. Therefore, the study participants followed a balanced protein distribution pattern, ensuring a more even distribution of protein intake at each meal. All meals were eaten under supervision of a registered dietician and controlled for energy and macronutrient content. Breakfast (~7:00 h), lunch (~12:00 h), and dinner (~17:00 h) were consumed no more than ~5 h apart to support muscle protein synthesis [18,28]. The testing and training sessions were performed at ~18:45 h. The participants ingested 0.5 g kg^−1^ day^−1^ whey protein isolate or flavor-matched maltodextrin ~30 min before bedtime (~23:00 h). The food intake 24 h prior to the two baseline assessment and the posttest were exactly matched. Water was provided *ad libitum*.

### 2.8. Statistical analysis

To calculate sample size and run statistical analysis, G*Power software [29] and SPSS version 25.0 (IBM Analytics, Armonk, NY) were used, respectively. Considering an effect size of 0.8, an alpha level of 0.05, and a beta level of 0.08, the initial calculation suggested that a minimum of six participants per group would be necessary. However, to accommodate for possible participant dropout during the data collection phase, the sample size was subsequently augmented to eight participants per group. Results were presented as mean ± *SD*. Shapiro-Wilk’s test checked normality in the data distribution. A three-way mixed linear model (treatment × time) with the treatment (PRO and CHO) as between factors and acute response in different time points [resting, immediately after completion, and 45 min post-SIT (early response), and 24 h as well as 48 h post-SIT (late response)], and training session (initial vs. final session) as the within factors compared the collected data. The level of α was set at 0.05.

## 3. Results

### 3.1. Diet control

As shown in Table 3, there was no between-group difference in the composition of macronutrients (i.e. excluding the intervention beverages) in the standardized diet (*p* > 0.05). After the training intervention, the results showed that only 10 percent (one participant) in the PRO group accurately reported consuming protein. Meanwhile, the remaining participants (90%) stated that they were unaware of what they had ingested. In the CHO group, only 20 percent (two participants) correctly identified that they had eaten carbohydrates, and the remaining participants (80%) reported not knowing what they had consumed.

**Table 3.** Standardized diet.

	Groups		*p*
	PRO	CHO	
Energy (Mj day^−1^)	10.3 ± 0.2	10.2 ± 0.3	0.95
Protein (%energy)	17.1 ± 0.2	17.2 ± 0.3	0.51
Protein (g day^−1^)	105.8 ± 4.3	106.7 ± 3.8	0.67
Carbohydrate (%energy)	56.3 ± 0.7	56.1 ± 0.3	0.18
Carbohydrate (g day^−1^)	346.3 ± 7.1	349.0 ± 17.4	0.92
Fat (%energy)	20.0 ± 0.0	20.0 ± 0.0	1.00
Fat (g day^−1^)	54.2 ± 1.0	54.9 ± 1.3	0.26

Values are means ± SD.

### 3.2. Physiological and performance parameters

The mean values of the physiological parameters and swimming performance of the participants are presented in Table 4. The mentioned variables showed no between-group difference at the baseline. VO_2_peak, vVO_2_peak, PPO, and MPO were significantly (*p* < 0.05) enhanced over time in both PRO (3.7%, 2.3%, 3.1%, 3.3%, respectively) and CHO (2.7%, 3.2%, 4.9%, 2.7%, respectively) groups. No between-group difference was found in the magnitude of the changes in the mentioned variables over time (*p* > 0.05). Interventions didn’t affect VO_2_/HR, HR_max_, VT_1_, VT_2_, and 200-m swimming performance (*p* > 0.05).

**Table 4.** Physiological and performance adaptations to interventions.

	PRO				CHO			
	Pre-test	Post-test	*P*	*ES*	Pre-test	Post-test	*P*	*ES*
VO_2_peak (ml kg^−1^ min^−1^)	53.0 ± 4.7	§55.0 ± 3.7	.002	0.51	51.1 ± 5.0	§52.5 ± 5.2	.007	0.37
vVO_2_peak (km min^−1^)	1.27 ± .06	§1.30 ± .05	.011	0.54	1.24 ± .04	§1.28 ± .03	.007	0.61
VO_2_/HR (ml beat^−1^ min^−1^)	2.9 ± 2.7	21.1 ± 2.1	.676	0.08	21.4 ± 2.5	21.8 ± 1.7	.283	0.18
HR at VO_2_peak (%max)	88.7 ± 2.3	89.1 ± 1.9	.612	0.18	88.0 ± 3.5	88.6 ± 2.3	.329	0.21
VT_1_ (%VO_2_peak)	75.4 ± 3.4	75.9 ± 2.5	.516	0.16	78.0 ± 4.3	78.6 ± 4.2	.529	0.14
VT_2_ (%VO_2_peak)	87.6 ± 2.5	88.0 ± 2.7	.874	0.15	86.7 ± 3.2	87.2 ± 3.0	.407	0.16
PPO (W)	212.0 ± 22.2	§218.6 ± 19.7	.024	0.31	219.6 ± 22.2	§23.5 ± 26.4	.008	0.44
MPO (W)	141.0 ± 13.4	§145.7 ± 15.8	.013	0.32	146.1 ± 1.6	§15.0 ± 1.5	.022	0.37
200-m swimming (s)	129.3 ± 13.7	129.0 ± 13.8	.100	0.07	128.2 ± 11.6	127.9 ± 12.2	.711	0.02

Values are means ± SD.

VO_2_peak, peak oxygen uptake; vVO_2_peak, velocity at VO_2_ peak, peak oxygen uptake; VT_1_, first ventilatory threshold; VT_2_, second ventilatory threshold; HR, heart rate; PPO, peak power output; MPO, mean power output.

§Significantly greater than pre-training value (P < 0.05).

### 3.3. Creatine kinase responses

**Figure 2.**
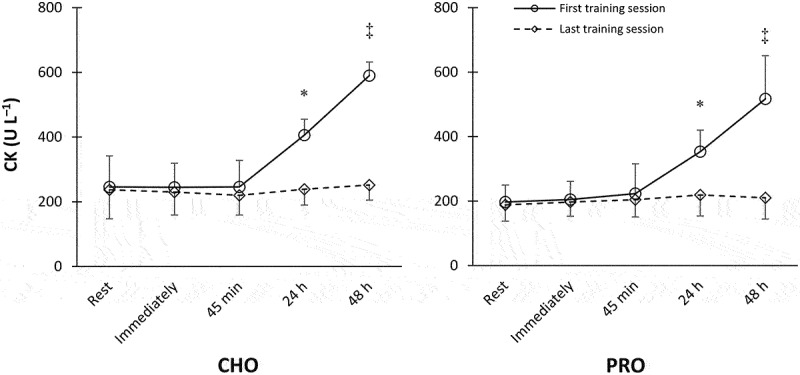
Creatine kinase concentrations after the first and last training sessions. *N* = 10 for each group. *Significantly greater than resting values. ‡ Significantly higher than all other time points (*p* < 0.05).

No between-group difference was found at the baseline (*p* = 0.482). In both PRO and CHO groups and only after the first session, CK concentrations elevated from rest to 24 h (*p* = 0.008 and 0.0003, respectively) and from 24 h to 48 h (*p* = 0.005 and 0.00007, respectively), with a significant increase at 48 h that exceeded all previous time points (*p* < 0.005) (Figure 2).

### 3.4. Cytokines response

**Figure 3.**
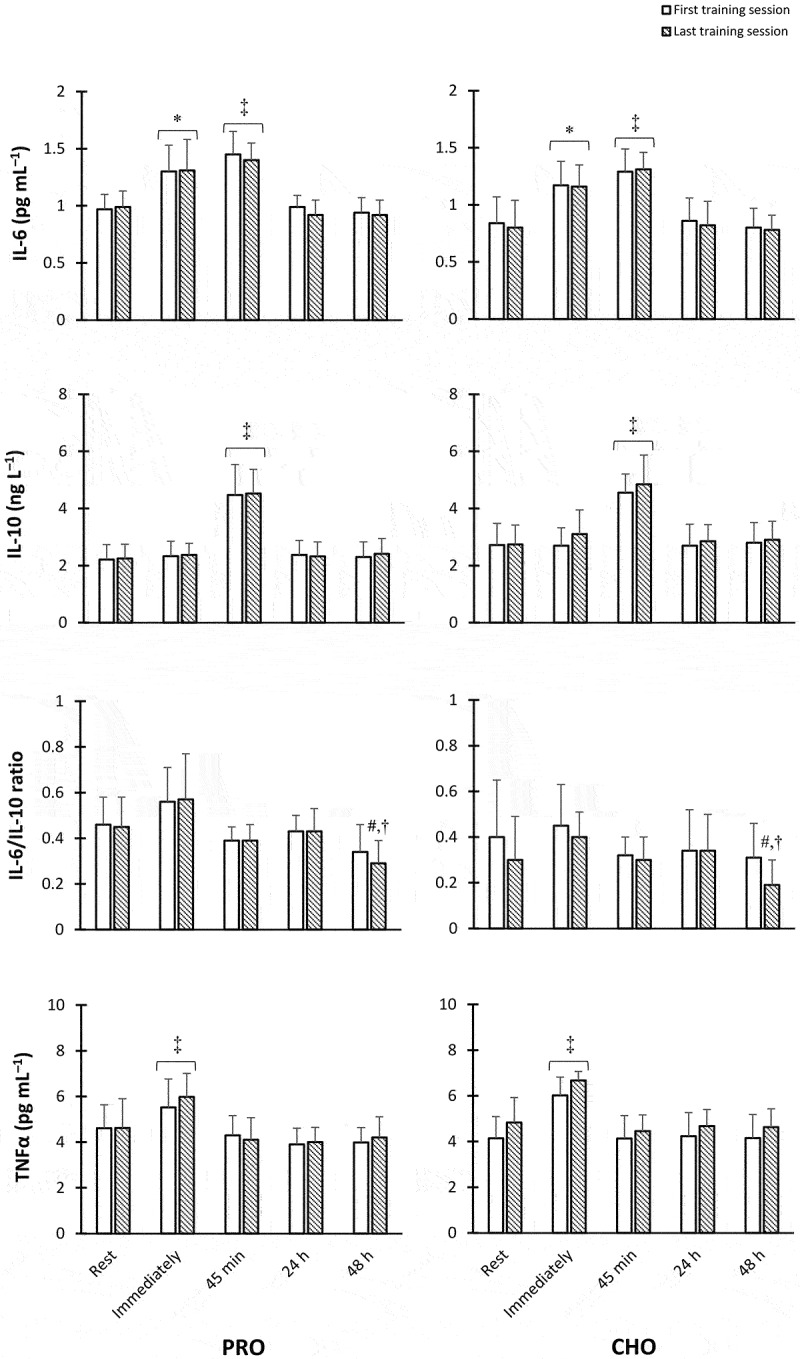
Concentrations of (a) Interleukin-6 (IL-6), (b) IL-10, (c) IL-6/IL-10 ratio, and (d) tumor necrosis factor alpha (TNFα) in both PRO and CHO groups. *Significantly greater than resting values. ‡ Significantly higher than all other time points. #Significantly lower than rest and immediately after SIT in the first session. †Significantly lower than rest and immediately after SIT in the last session (*p* < 0.05).

No between-group difference was observed at the baseline for inflammatory cytokines [IL-6 (*p* = 0.334), IL-10 (*p* = 0.498), IL-6/IL-10 ratio (*p* = 0.558), and TNFα (*p* = 0.353)]. In both PRO and CHO groups, IL-6 concentrations elevated immediately following the first session of SIT (*p* = 0.004 and 0.01, respectively) and the last session of SIT (*p* = 0.01 and 0.005, respectively). Also, IL-6 levels enhanced further 45 min after the first session of SIT (*p* = 0.0003 and 0.0001, respectively) as well as the last training session (*p* = 0.001 and 0.0005, respectively) when compared to baseline values; however, its levels returned to resting values after 24 h and remained unchanged until 48 h post-exercise. In both PRO and CHO groups, IL-10 concentrations remarkably increased 45 min after the first and last training session compared to the rest of the time points (*p* < 0.0001) and returned to pre-training values 24 h post-SIT. 48 h after the final SIT session, the ratio of IL-6 to IL-10 was significantly lower than its values at the time points of rest and immediately after SIT in both the first and last sessions in both groups (*p* < 0.04). In both PRO and CHO groups and the first and last training sessions, TNFα levels significantly elevated from rest to immediately after SIT (*p* < 0.001) and returned to values equivalent to the baseline afterward (Figure 3).

## 4. Discussion

The present study examined the effect of a single session (acute) and multiple sessions performed over two weeks (chronic) swimming SIT on responses of inflammatory cytokines and muscle damage markers and if increasing protein intake through protein supplementation before sleep may affect these responses. The primary finding of this experiment was that swimming SIT reduced the concentrations of exercise-induced markers of muscle damage and shifted the post-SIT (48 h) response of cytokines to an anti-inflammatory status. This effect was independent of protein supplementation, as whey protein consumed before sleep failed to reduce blood markers of muscle damage and failed to induce systemic anti-inflammatory status. To the best of our knowledge, this study is the first to indicate a swimming SIT with short intervals performed over two weeks shifts the circulatory cytokines to an anti-inflammatory status.

Regarding muscle damage markers, our findings are contrary to previous studies indicating the positive impact of protein intake immediately around training sessions on ameliorating sarcolemma disruption markers [30]. The time of the protein supplementation is the main difference between our experiment and previous studies. Our results support Larsen and colleagues [10], who indicated that over a week of endurance-type intensive training, elevation in circulatory markers of muscle damage is not alleviated by pre-sleep whey protein intake compared to isocaloric carbohydrate ingestion. Likewise, other studies [6,31] have shown that consuming protein before sleep, regardless of protein resource (whey or plant-based protein), failed to facilitate muscle recovery following damaging eccentric exercise. Although Thomson and colleagues [30] showed the benefits of pre-sleep protein supplementation on recovery from endurance training, their protein ingestion was still immediately after the exercise, indicating the importance of protein timing. To support this, Levenhagen and colleagues [32] have suggested that protein supplementation immediately after a 60-min endurance causes more remarkable whole-body protein synthesis compared to protein ingested 3 h after the training.

Consistent with previous research, our results indicated no positive effects of pre-sleep protein supplementation on inflammatory biomarkers [6,26]. Levels of IL-6 significantly elevated following SIT in both PRO and CHO groups, which aligns with previous findings [5,33]. Ferreira and colleagues [5] have shown that a 2-week SIT (13 × 30 s *all-out* sprints with 15 s relief between efforts) with prior caffeine intake induces an increase in serum IL-6, reaching its maximal levels 45 min after SIT. Gerosa-Neto and colleagues [33] demonstrated that in response to high-intensity interval training (10 × 60 s at 100% velocity associated with VO_2_max), concentrations of IL-6 remained elevated up to 60-min after the acute training session. Elevated levels of IL-6 detected immediately after exercise can indicate muscle metabolism and are not exclusively a consequence of inflammation [34]. An example of how IL-6 functions are when muscle glycogen is depleted. This leads to an increase in the release of IL-6 from the muscle, which signals other tissues, such as adipose tissue, to release more circulating free fatty acids to supply energy substrates needed for the contracting skeletal muscle [35]. It has been shown that an increase in plasma IL-6 concentrations induced by whole-body exercise is attenuated with six weeks of intensive intervals (5 × 3 min at 90% VO_2_max [36]). Our results indicated although aerobic and anaerobic power increased in response to SIT, no change was observed in the SIT-induced increase in IL-6. Increased vVO_2_peak and power output after the training period causes to complete all-out SIT in higher intensities leading to an increase in metabolic demand compared to the first session. This complies with previous studies indicating that two weeks of SIT doesn’t affect IL-6 levels pre- to post-training [5]. IL-6 also serves as an anti-inflammatory factor because an increase in IL-6 following exercise stimulates IL-10 production, a known inhibitor of pro-inflammatory TNFα [37]. Per this and other studies [5,33], we observed a transient elevation in TNFα immediately after SIT, after that by an elevated IL-10 peaking 45 min after SIT. IL-6 and IL-10 showed a maximal level 45 min after SIT. In contrast, TNFα levels did not significantly differ from the resting levels, suggesting that a single swimming SIT session can elicit an IL-6 reaction consistent with its anti-inflammatory as well as metabolic effects [5].

Another important finding of this experiment was the decrease in the late IL-6/IL-10 ratio (48 hours after exercise) following a 2-week swimming SIT program, along with the absence of any changes in CK concentration. Our findings support Ferreira and colleagues [5], who reported that six sessions of cycling SIT performed over two weeks can switch the late post-exercise cytokine response (24 and 48 h after exercise) to an anti-inflammatory profile and prevent the late increase in CK. Longer time expended at maximal oxygen uptake through more sprints and shorter recovery [5] as well as anti-proteolytic effects of exercise [38] are possible explanations for improving cytokine status and lack of change in CK concentrations, respectively. Two weeks of swimming SIT was also associated with improvements in peak oxygen uptake and upper-body anaerobic power. Rodas [39] indicated that performing a 2-week SIT consisting of 2–7 bouts of 15 s *all-out* cycling plus 2–7 bouts of 30 s *all-out* cycling with 45 s recovery between efforts improves VO_2_max through the enzymatic activity of energetic pathways. Ferreira and colleagues [5] reported that two weeks of cycling SIT with more sprints and shorter recovery is an effective stimulus to enhance aerobic power. The enhanced discharge rate of high-threshold motor units [40] and the increased muscle PCr concentration [39] are potential reasons for the improved power output observed.

One limitation of this study was the inability to monitor sleep closely, and another was the inclusion of only male athletes. This makes it difficult to generalize our results to female runners. Larsen and colleagues [10] have mentioned that levels of CK may be attenuated by the presence of estrogen in the circulation following muscle-damaging exercise. The adaptations observed in our study are specific to our regimens, and it is unclear whether similar changes would occur with different SIT protocols and nutritional strategies. Another limitation is the relatively short duration of the study. A brief study duration may not allow for a comprehensive evaluation of the intervention’s long-term effects or sustained impact. It is possible that a more extended period could reveal different outcomes or provide a more thorough understanding of the intervention’s effectiveness. Also, the study relied on indirect plasma markers to assess certain variables of interest. While these markers provide valuable insights, they may not directly reflect a complete picture of the observed effects.

## 5. Conclusions

Swimming SIT attenuates exercise-induced CK and shifts the post-exercise (48 h) status of cytokines to an anti-inflammatory profile. We provided trained swimmers with either a PRO or a CHO beverage of equal caloric value every night preceding going to bed while maintaining a standardized diet for as short as two weeks. Consumption of whey protein before sleep did not result in a decline in blood factors related to muscle damage or induce a systemic anti-inflammatory response.
